# Effects of field plot size on prediction accuracy of aboveground biomass in airborne laser scanning-assisted inventories in tropical rain forests of Tanzania

**DOI:** 10.1186/s13021-015-0021-x

**Published:** 2015-05-07

**Authors:** Ernest William Mauya, Endre Hofstad Hansen, Terje Gobakken, Ole Martin Bollandsås, Rogers Ernest Malimbwi, Erik Næsset

**Affiliations:** 1 Department of Ecology and Natural Resource Management, Norwegian University of Life Sciences, P.O. Box 5003, Oslo, NO 1432, Ås Norway; 2 Department of Forest Mensuration and Management, Sokoine University of Agriculture, P.O. Box 3013, Morogoro ᅟ, Tanzania

**Keywords:** Airborne laser scanning, Model-assisted estimation, Plot size, Aboveground biomass

## Abstract

**Background:**

Airborne laser scanning (ALS) has recently emerged as a promising tool to acquire auxiliary information for improving aboveground biomass (AGB) estimation in sample-based forest inventories. Under design-based and model-assisted inferential frameworks, the estimation relies on a model that relates the auxiliary ALS metrics to AGB estimated on ground plots. The size of the field plots has been identified as one source of model uncertainty because of the so-called boundary effects which increases with decreasing plot size. Recent research in tropical forests has aimed to quantify the boundary effects on model prediction accuracy, but evidence of the consequences for the final AGB estimates is lacking. In this study we analyzed the effect of field plot size on model prediction accuracy and its implication when used in a model-assisted inferential framework.

**Results:**

The results showed that the prediction accuracy of the model improved as the plot size increased. The adjusted R^2^ increased from 0.35 to 0.74 while the relative root mean square error decreased from 63.6 to 29.2%. Indicators of boundary effects were identified and confirmed to have significant effects on the model residuals. Variance estimates of model-assisted mean AGB relative to corresponding variance estimates of pure field-based AGB, decreased with increasing plot size in the range from 200 to 3000 m^2^. The variance ratio of field-based estimates relative to model-assisted variance ranged from 1.7 to 7.7.

**Conclusions:**

This study showed that the relative improvement in precision of AGB estimation when increasing field-plot size, was greater for an ALS-assisted inventory compared to that of a pure field-based inventory.

## Background

Tropical forests play an important role in the global carbon cycle as they store about 40% of the global terrestrial carbon, and absorb larger amounts of CO_2_ from the atmosphere than any other vegetation type [1]. Despite their potential, tropical forests continue to be exploited at alarming rates, by being converted into secondary forest and many other forms of land use. In an effort to conserve tropical forests, the United Nations Framework Convention on Climate Change (UNFCCC) has developed the mechanism called Reducing Emissions from Deforestation and Forest Degradation in tropical countries (REDD+). There is high interest in seeing such initiatives to take form, but a key limitation for successful implementation of REDD+ is reliable methods for quantifying forest aboveground biomass (AGB) [2,3]. Such methods are important because payments for carbon offsets under REDD+ are based on estimates of carbon stock and stock changes over time. Moreover, AGB information is also useful for understanding the contribution of the tropical forests to the global carbon cycle and ecosystem processes [4].

Airborne laser scanning (ALS) has emerged as one of the most promising remote sensing technologies to support AGB forest inventories in boreal-, temperate-, and tropical forests [5]. A particular strength of ALS for forest applications is its ability to accurately characterize the three-dimensional (3D) structure of the forest canopy [6]. Such information is more useful for forest inventories than the information from other remote sensing techniques see e.g. [7]. Height and density metrics derived from the ALS data has been reported to be highly correlated with AGB see e.g. [8,9]. Furthermore, ALS has shown to be superior to other remote sensing data sources because the relationship between AGB and the remotely sensed information has a much higher saturation level for ALS compared to other types remote sensing. Because of this, ALS is a highly appropriate choice of technique in high-biomass forests. Based on its potential, ALS has recently been recommended for Monitoring, Reporting and Verification (MRV) systems under REDD+ initiatives [10].

Estimation of AGB using ALS is often carried out according to the area-based approach (ABA) [11]. In ABA, empirical models between various metrics derived from the ALS data and AGB values obtained in geo-referenced field sample plots are fitted. The area of interest is then tessellated into grid cells [12] with the same size as the plots [13,14] and the developed models are used to provide cell-wise predictions of AGB. Finally, estimates for the particular area of interest (forest stand, forest property, village, district, or nation) are provided by summing the individual cell predictions. For some estimation approaches, adjustment of model prediction bias [15] is also carried out.

As indicated above, the modeled relationship between ALS metrics and ground-based values is of fundamental importance for the outcome of the ALS-assisted estimation. The use of field plot data for model development requires co–registration of field plot location with the ALS data [16,17]. In an ALS-assisted inventory, the point cloud is extracted only within the plot perimeter. However, in field measurements trees are treated as being inside plots if the center point of the stem is inside the plot. This is a challenge in ALS-assisted forest inventory, since the crowns of trees just outside the plot border partly extend into the plot area which means that the ALS data will be affected by trees that are not registered in field. Conversely, also trees just inside the plot extend their crowns beyond the plot boundary. This means that there may be mismatch between the data captured in field and from the air.

In order to reduce these boundary effects, it has been suggested in a number of studies to use larger plots in ALS-assisted forest inventory see e.g. [18,19]. This is because, as plot size increases, the perimeter to area ratio decreases and thus the plots include a lower proportion of boundary-related elements. Similarly, the relative and negative influence of a given plot positioning error is reduced because the relative overlap between the field- and ALS-data becomes larger as plot size increases. Reduction in model errors are also expected by increasing plot size due to so-called spatial averaging of the errors [20], because both the field observations and the ALS data capture more of the spatial variation as they increase in size. Thus, as plot sizes increase, the variances of field-based and ALS-assisted estimates are expected to be reduced, which means that fewer plots are needed to reach a certain precision of an AGB estimate. However, large plots also have disadvantages by being more complicated to measure, which may affect the time consumption for collecting field measurements [21], This makes it challenging to select the “optimal” plot size that balances the tradeoff between plot size, sample size (number of plots), on-plot costs, traveling costs and precision of ALS-assisted AGB estimates in different forest types.

As indicated above, plot size has a profound effect on the precision of ALS-assisted AGB estimates for several reasons. Likewise, the plot size has an impact on the precision of pure field-based estimates for reasons mentioned above; larger plots capture more of the variability in the area of interest and thus precision will tend to improve as long as the sample size is kept constant. A key question is therefore if larger plots will favor ALS-assisted estimation precision to the same extent as it favors field-based estimation precision. Different responses to plot size should have a direct impact on how tropical ALS-assisted field sample surveys should be designed as their designs currently are “optimized” for pure field-based estimation.

Forest sample surveys are often designed according to design-based (probability-based) principles. Simple random sampling is one of these principles, and analytical and so-called design-unbiased estimators and corresponding variance estimators exist for a great number of such designs. When auxiliary data such as those acquired by ALS are at hand for the entire area of interest, or at least with partial coverage of the area of interest, use of these data can greatly improve the precision over a pure field-based estimate assuming the same design. The inferential framework applied under probability sampling when a model is used to predict AGB using the ALS data is known as design-based model-assisted (MA) estimation. In the MA framework, the model is used to predict AGB for grid cells and then AGB is summed over all grid cells as indicated in the ABA, but in addition to that, the model predictions for the ground samples are used to provide an estimate of bias in the model predictions, which corrects the pure model-based estimate. Several studies see e.g. [22-24] have indicated the potential of MA estimation in reducing the variance of AGB estimates in boreal forests, but apart from some indications provided by [23], neither of them has analyzed how the variance of the estimates is affected by changes in field plot sizes. In tropical forests where the current study was conducted, there is even less knowledge regarding performance of MA estimation using ALS with varying plot sizes. Several tropical studies have examined the effects of plot size on model prediction accuracy See e.g. [25-27], but none of them have assessed the effects on the precision of AGB estimates and compared such precision estimates with corresponding precision of field-based AGB estimates using the same sampling design, which is of fundamental importance for designing future sample surveys serving multiple purposes and estimation approaches.

The objectives of this study were to (1) examine the effects of field plot size on AGB regression model quality, (2) assess plot boundary effect and its impact on model quality based on the field data, and (3) quantify the precision of ALS-assisted estimates of AGB relative to field-based estimates of AGB assuming the same design for different plot sizes. The study was conducted in tropical rain forest in Tanzania with high AGB densities, which was expected to represent a particular challenge in terms of large boundary effects.

## Results

### Effects of field plot size on ALS AGB predictions

**Figure 1:**
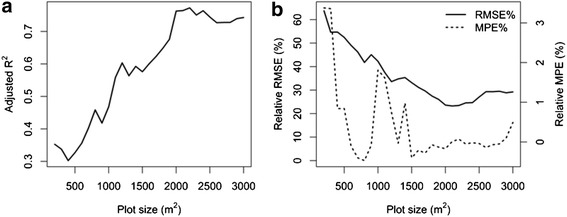
Model quality and plot size. **(** **a** **)** Adjusted R^2^ versus plot sizes. **(** **b** **)** Relative MPE% and RMSE% versus plot sizes.

**Figure 2:**
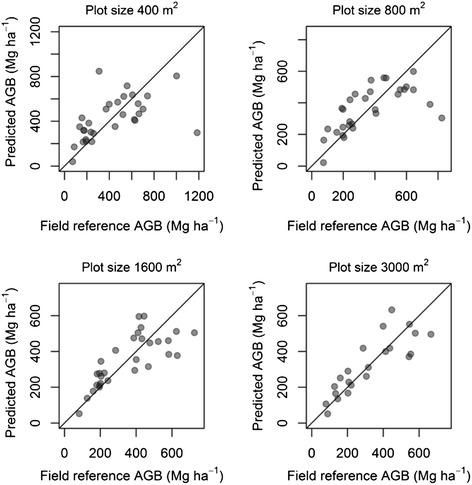
Relationship between field reference AGB and predicted AGB for different plot sizes.

To assess the effect of plot size on ALS assisted forest inventory, we first fitted the regression models for each of the plot sizes. The independent variables selected varied between the models developed for the different plot sizes (Table 1). The number of variables varied between two and three. For all models, the parameter estimates were significantly different from zero (*p* < 0.05) and the VIF values were <10, indicating acceptable levels of multicolinearity. The variability explained by separate models (i.e. adjusted R^2^) improved as the plot size increased, with few exceptions (Figure 1a). The adjusted R^2^ ranged from 0.35 for the plot size of 200 m^2^ to 0.74 for the plot size of 3000 m^2^. The RMSE% values for LOOCV decreased non-linearly with increasing plot size, from 63.8 to 29.2% (Figure 1b). The MPE% values (Figure 1b) and the pattern of under predictions for plots with high AGB were relatively lower for larger plots compared smaller (Figure 2). However, it should be noted that the number of the larger plots was relatively small.

**Table 1:** **Selected ALS metrics for different plot sizes**

**Plot size (m** ^**2**^ **)**	***n***	**Selected variables** ^**a**^		
200	30	D_0_.F	D_1_.L	log.H_80_.F
300	30	D_1_.L	log.H_90_.F	log.D_0_.L
400	30	H_80_.F	D_1_.L	
500	30	H_70_.F	D_1_.L	
600	30	H_70_.F	D_1_.L	
700	30	H_90_.F	D_1_.L	
800	30	H_90_.F	D_1_.L	
900	30	H_90_.L	D_1_.L	
1000	30	Hsd.L	D_1_.L	log.D_0_.F
1100	30	D_3_.F	D_2_.L	log.H_10_.F
1200	30	H_mean_.F	D_1_.L	
1300	30	H_70_.F	D_1_.L	
1400	30	D_3_.F	D_2_.L	log.H_10_.F
1500	30	H_70_.F	D_1_.L	
1600	30	H_60_.F	D_1_.L	
1700	30	H_60_.F	D_1_.L	
1800	30	H_60_.F	D_1_.L	
1900	30	H_60_.F	D_1_.L	
2000	25	H_60_.F	D_1_.L	
2100	25	H_60_.F	D_1_.L	
2200	24	H_60_.F	D_1_.L	
2300	24	H_60_.F	D_1_.L	
2400	24	H_70_.F	D_1_.F	
2500	22	H_70_.F	D_1_.F	
2600	22	H_70_.F	D_1_.F	
2700	22	H_70_.F	D_1_.F	
2800	22	H_70_.F	D_1_.F	
2900	22	H_70_.F	D_1_.F	
3000	22	H_60_.F	D_1_.F	

^a^D_0_.F, D_1_F.and D_3_.F = Canopy densities corresponding to the proportion of first echoes above fraction #0 (2 m), #1 and #3 (see text). ^a^D_0_.L, D_1_.L and D_2_L. = Canopy densities corresponding to the proportion of last echoes above fraction #0 (2 m), #1 and #2 (see text).

H_10_.F, H_60_.F, H_70_.F, H_80_.F and H_90_.F. = ALS height percentiles of the canopy height for the first echo.

Hsd.L = Standard deviation of the canopy height of the first echoes.

H_mean_.F = Arithmetic mean of the first echo ALS canopy height.

### Boundary effects

Boundary effects were studied by analyzing how the relative residual errors of the models were affected by the ground reference AGB of the trees in an outer buffer zone for different field plot sizes. Our results showed that SAGB_buffer_ and MAGB_buffer_ contributed to explaining the variation in the relative residual errors (Table 2). Relating the absolute value of the relative residual with plot size using simple linear regression model indicated that there was a highly significant effect of plot size (*p* < 0.0001). Furthermore, the parameter estimate for plot size was negative showing that the relative residual is larger in absolute terms for small plots compared to larger plots (Table 3).

**Table 2:** **Coefficient estimates for models explaining residual errors of AGB using information extracted from buffer zones**

**Models** ^**1**^	**Model parameter**	**3 m buffer**			**6 m buffer**		
		**Parameter estimate**	**p-value**	**AIC**	**Parameter estimate**	**p-value**	**AIC**
Model 1	Intercept	−0.0159	0.8022	297	−0.1835	0.0206	654
	^2^SAGB_buffer_	0.0838	<0.0001		0.1892	<0.0001	
Model 2	Intercept	−0.0321	0.5826	266	0.0501	0.4674	663
	^2^MAGB_buffer_	0.4865	<0.0001		0.7015	<0.0001	

^1^Models = Two models; Model 1 uses SAGB_buffer_ as fixed effects with plot identity as random effect. Model 2 uses MAGB_buffer_ with plot identity as random effect (see text).

^2^SAGB_buffer_ = Ratio of either sum of AGB at the buffer to the ground reference AGB per hectare, MAGB_buffer_ = ratio of Maximum AGB at the buffer to the ground reference AGB per hectare (see text).

**Table 3:** **Parameter estimates for the model relating relative residual in absolute form and plot sizes**

**Coefficients**	**Parameter estimates**	**p-value**
Intercept	0.5060	<0.0001
Plot size	−0.0002	<0.0001

### Efficiency of ALS-assisted AGB estimation

**Figure 3:**
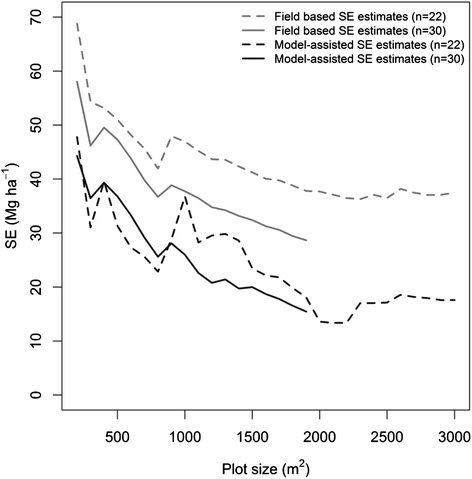
Field-based and model-assisted SE estimates for different plot sizes covered in two sample datasets (i.e. 200 to 1900 and 200 to 3000 m^2^).

**Figure 4:**
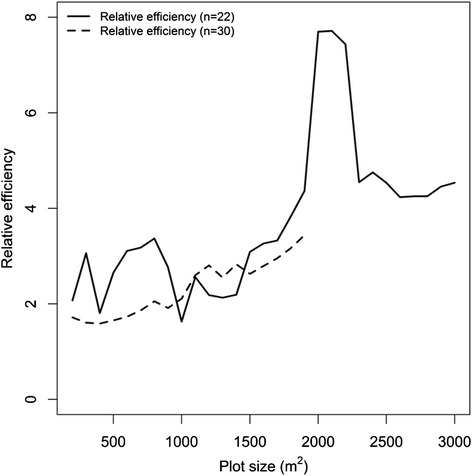
Relative efficiency for different plot sizes.

The SE estimates for the field-based AGB estimates were larger than the corresponding model-assisted SE estimates (Figure 3). For the plot sizes that allowed consistent analysis for all 30 sizes, i.e. from 200 to 1900 m^2^, the field-based SE estimates decreased from 58.0 Mg ha^−1^ to 28.7 Mg ha^−1^, while the model-assisted SE estimates decreased from 44.3 Mg ha^−1^ to 15.5 Mg ha^−1^. Relative to the mean of field reference AGB for the plot size from 200 to 1900 m^2^, the field –based SE estimates decreased from 14.1% to 8.2% , while for the model-assisted estimates decreased from 10.8% to 4.4%. Similarly, for the larger plots (up to 3000 m^2^) for which 22 observations were available for consistent analysis, the SE estimates for model-assisted were relatively much smaller compared to the field-based inventory. In both cases the SE was higher for smaller plots compared to the larger plots. Generally, the effectiveness of the ALS-assisted estimates was more improved as the plot size increased compared to the field-based estimates. This indicates that larger plots are relatively more favorable for ALS-assisted estimation than for pure field-based estimation. The RE values were >1 with a maximum value of 3.4 (Figure 4) for the plot sizes ranging from 200–1900 m^2^ for which we have a complete dataset of 30 plots. For the other set with plot size up to 3000 m^2^ the maximum RE value was 7.7. It should be noted that the peak in relative efficiency for the smallest dataset (22 plots) in Figure 4 was caused by considerable change in the observed AGB for a single plot when increasing the plot size beyond 2000 m^2^. The increasing AGB was due to a large tree that was included in the plot measurements once the plot radius exceeded 25 m. This illustrates that in a small dataset the results can be sensitive to individual observations and even to the presence of individual trees.

## Discussion

The findings of this study demonstrated the importance of choosing appropriate field plot sizes in ALS-assisted forest inventories in tropical forests. This is particularly important given that field campaigns are expensive and time consuming, and linking field measurements with remotely sensed data in the most effective manner would benefit both REDD+ implementations, together with all other studies related to forest carbon cycle. The current study extends previous research conducted in tropical forests, by having a dataset with a wide range of plot sizes. Furthermore, most of the previous studies have used rectangular plots.

See e.g. [18,26], whereas in this case circular plots have been used. Circular plots are more convenient for remote sensing studies compared to square or rectangular plots because only a single coordinate together with a plot radius are needed to match the two data sources geographically [19,28,29]. Circular plots are also within certain sizes easier to establish in the field because they have one dimension (i.e. radius) that defines the plot boundary. The use of circular plots minimizes the plot boundary effects because of a smaller circumference to area ratio than all other plot shapes. However, the visibility from the plot center to the perimeter on a circular plot is increasingly hampered as the plots get larger, which increase per tree measurement time for the border trees. An increase of the area of a rectangular plot would not necessarily mean increased marginal cost (cost of including one more tree) if the width of the plot is kept constant and inclusion of trees are made with reference to the long side. However, rectangular plots are in general more difficult to establish. For example, in rugged terrain it can be difficult to keep the sides parallel.

Our findings demonstrated empirically the positive effects of increasing plot sizes on improved predictive power of the AGB models. The model fit (adjusted R^2^) of the regression models was improved as plot size increased. Reduced circumference to area ratio, spatial averaging, and less effect of positioning errors are probably the main reasons. The fit of our models are in line with previous ALS-based studies in both tropical forests and temperate forests. For example, [30] reported R^2^ of 0.78 in the tropical rainforest of Hawaii islands while [31] reported R^2^ of 0.64 in a tropical rainforest of West Africa. Furthermore, results from the cross-validation showed smaller RMSE% and MPE% (Figure1b) for larger plots compared to smaller plots. Similar trends have been reported and discussed by other authors in both temperate and tropical forests see e.g. [32].

Plot boundary effects have been discussed in previous studies see e.g. [16,33] as one among the sources of model error in ALS-assisted inventories, particularly when relying on small plots. We demostrated this in two steps; first by relating relative residuals to the sum of AGB per hectare for all trees in the buffer (SAGB_buffer_ ) and the maximum AGB per hectare for the largest tree in the buffer (MAGB_buffer_ ) where we noted that their importance were depending on the size of the buffer. The buffer conditions as expressed both by (MAGB_buffer_) and (SAGB_buffer_), seemed to have more impact on the residual error with decreasing distance to the plot judged by the AIC values (Table 2), which is logical. Furthermore, when comparing the two variables, SAGB_buffer_ seemed to lose less explanatory power by going from 3 meter to 6 m buffer than MAGB_buffer_. This result was also expected because the represetation of the whole buffer by SAGB_buffer_ is less prone to be changed by the increase in size compared to MAGB_buffer_ which is calculated from a single tree. Furthermore, the decrease in ALS model residuals (Table 3) with increasing plot sizes is a clear indication that smaller plots are more prone to boundary effects compared to larger plots.

Contribution of ALS data in improving precision of AGB estimates was also demonstrated within varying ranges of plot sizes. The RE values were > 1, indicating that ALS-assisted estimation is more efficient compared to pure field-based estimation. To achieve similar precision of a pure field-based estimate relying on simple random sampling, would mean to increase the sample size for the field-based inventory by a factor equivalent to the value of RE, which would have a substantial effect on field inventory costs. In general, the gain in relative efficiency was more pronounced as plot size increased, suggesting that larger plots are more favorable when ALS-data are used to assist in the estimation. Even though we did not undertake any analysis of cost-efficiency, the trend would be toward larger and fewer plots as one introduces ALS to support in the estimation. Even this finding can be attributed to the effects discussed above, namely reduced boundary effects and co-registration errors.

Despite the potential of improving the efficiency of ALS-assisted inventories by use of larger plots, choice of an “optimal” plot size must be seen in a broader context by considering a number of factors including; sample sizes, on-plot costs, traveling costs and overall field inventory design. Several authors see e.g. [20,23,30] have indicated that selection of the plot size also will depend on forest types, available resources and the needed precision. Based on our findings, there is larger potential of gaining efficiency of using ALS data in this type of forest when the field plot size is larger than 1200 m^2^. Finally, even though our study was limited to the tropical rainforests of Tanzania, the major findings are of interest and efforts should be taken to upscale to other tropical forests by considering more factors that would lead to selection of “optimal” plot size.

## Conclusions

To conclude, our study has demonstrated that field plot size effect the prediction accuracy of ALS-assisted AGB estimation in the tropical forests. Generally, there was substantial improvement in prediction accuracy from larger plots compared to smaller plots. Indicators of boundary effects were also identified and confirmed to have significant effects on the model quality. From a purely technical point of view, our results suggested that it is relatively more favorable to increase the plot size when ALS is used to enhance the estimates. This study showed that there is a relative improvement in precision of ALS-assisted AGB estimation, compared to pure field-based estimation up to around 3000 m^2^ in this type of forest. However, the maximum plot size of 3000 m^2^ in the current study leaves an open question as to whether there are any additional gains in relative precision beyond this size. Future studies should be conducted to quantify the contribution of ALS to improve estimation precision for even larger plots as the basis for design of future inventories in tropical rainforests. Similar studies should also be conducted in other types of tropical forests.

## Methods

### Site description

**Figure 5:**
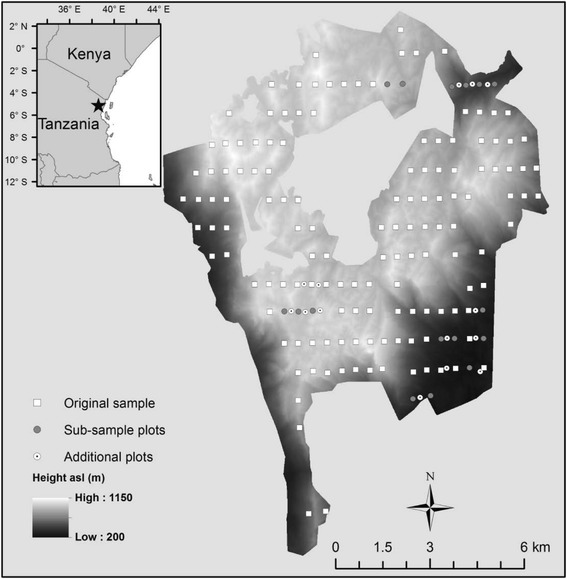
Study area and field plots layout. *Left:* Location of Amani nature reserve (marked with star). *Right:* Map of Amani nature reserve and the two samples of field plots.

The study was conducted in Amani nature reserve (ANR), which is situated in the southern part of the East Usambara Mountains in northern Tanzania (Figure 5). It was gazetted in 1997 with a protected area of 8,380 ha. ANR lies between 5°14' - 5° 04' S and 38° 30' - 38°40' E, with an altitudinal range of 190 to 1130 m above sea level [34]. Rainfall is heavy at higher altitudes and in the southeast of the mountain, with an average of 1900 mm annually. The dry seasons are from June to August and January to March, but rainfall is frequent throughout the year. The mean annual temperature is 20.6°C [35].

### Data collection

#### Sampling design

An initial probability sample of 173 field plots with an average size of 900 m^2^ were established across ANR according to a systematic design (450 m × 900 m distance between plots) in 1999–2000 by a non-governmental conservation and development organization, Frontier Tanzania [34] (Figure 5). The plots were revisited and re-measured in 2008–2012. In order to analyse plot size effects on AGB estimates, a small sub-sample of 30 large plots was established. Measurements on the 30 plots were acquired in a separate campaign after completion of measurements of the large sample. Due to high travel costs and long walking distances in the very steep and rough terrain, establishing a probability sample of 30 large plots across the entire study area was cost-prohibitive. Instead we developed a sampling strategy by which we took advantage of the a priori knowledge of the distribution of AGB in the large probability sample and selected purposefully three sub-regions within the study area in which the initial plots were revisited. There is a strong altitude-dependent AGB gradient in the study area. It was therefore important to capture the altitude gradient in each of the three sub-regions in order to resemble the AGB distribution in the initial probability sample.

In the sampled sub-regions, we first selected 16 of the plots in the initial probability sample for measurement. We also established 14 new and additional plots along the grid-lines of the probability sample and located them exactly mid-way between two existing plots. Thus, the distance between our plots was 225 m rather than 450 m.

**Figure 6:**
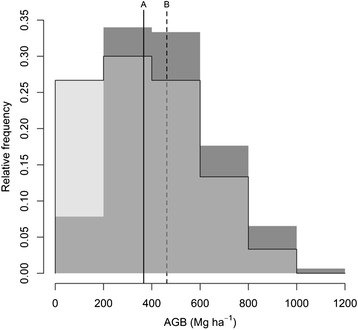
Distribution of AGB in the large probability sample (dark grey), in the small sample of 30 plots (900 m^2^) (light grey) and overlap between the two distributions (grey). The vertical line A indicates the mean of the small sample (366.0 Mg ha^−1^) and line B the mean of the large sample (461.9 Mg ha^−1^).

**Figure 7:**
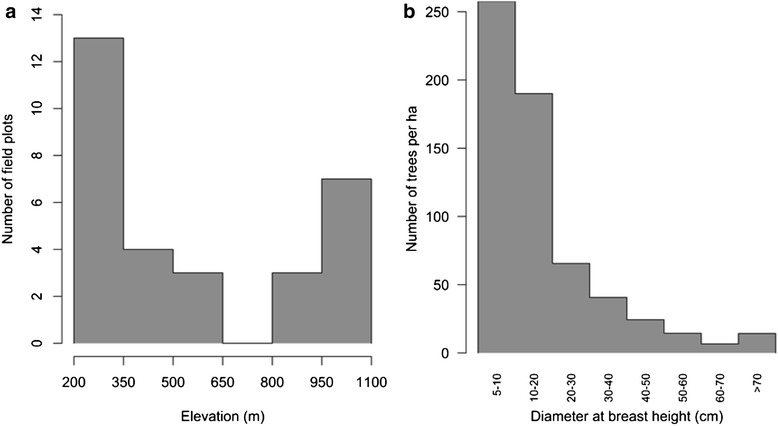
Distributions of field plots, elevation, number of trees per ha and tree sizes. **(** **a** **)** Number of field plots versus elevation. **(** **b)** Number of trees per ha versus tree sizes.

Although the resulting sample of 30 large plots was not selected according to probabilistic principles, it closely resembled essential properties of the large probability sample. First of all the AGB distributions of the two samples were similar (Figure 6). The mean AGB of the 30 plots with an area of 900 m^2^ was 366.0 Mg ha^−1^ (Table 4, Figure 6), while it was 461.9 Mg ha^−1^ for the large probability sample (Figure 6). The AGB range was 69.4-908.3 Mg ha^−1^ (standard deviation of 216.3 Mg ha^−1^) while it was 43.2-1147.1 Mg ha^−1^ (standard deviation of 214.7 Mg ha^−1^) for the large sample. Furthermore, the 30 plots covered an elevation range of 200 to 1000 m above sea level (Figure 7a) so that both the lowland forests (<800 m above sea level) and the sub mountain forests (>800 m above sea level) were represented. The 30 plots also covered a wide range of tree sizes (Figure 7b).

**Table 4:** **Summary of field data**

**Plot size (m** ^**2**^ **)**	**Number of plots ** **(n)**	**Mean ** **(Mg ha** ^**−1**^ **)**	**Standard deviation ** **(Mg ha** ^**−1**^ **)**	**Minimum ** **(Mg ha** ^**−1**^ **)**	**Maximum ** **(Mg ha** ^**−1**^ **)**
200	30	411.4	323.2	53.3	1179.5
300	30	401.0	257.3	48.2	816.0
400	30	424.5	275.8	72.6	1185.2
500	30	413.8	263.4	77.8	1148.0
600	30	395.3	243.9	87.9	1066.6
700	30	371.8	221.5	75.4	931.7
800	30	363.1	204.4	74.3	824.3
900	30	366.0	216.3	69.4	908.3
1000	30	367.1	210.1	62.4	859.7
1100	30	365.6	203.0	66.4	839.5
1200	30	365.0	193.7	78.4	797.6
1300	30	361.0	190.5	82.1	757.9
1400	30	352.3	184.7	87.3	707.0
1500	30	354.2	180.4	85.5	757.8
1600	30	353.2	174.1	82.2	725.5
1700	30	355.0	170.2	95.6	702.6
1800	30	355.9	163.9	91.5	696.5
1900	30	351.1	159.6	90.7	703.3
2000	25	352.2	170.8	89.6	669.3
2100	25	350.4	168.0	85.5	646.2
2200	24	344.7	169.3	89.3	631.1
2300	24	343.0	167.8	88.5	639.8
2400	24	344.2	171.3	87.9	677.7
2500	22	332.1	175.0	84.4	661.5
2600	22	334.1	183.1	91.8	669.9
2700	22	328.0	179.8	88.6	674.7
2800	22	322.7	177.7	85.4	665.9
2900	22	323.5	177.9	82.5	655.6
3000	22	321.0	179.7	79.7	666.7

Number of plots for the different plot sizes together with mean field reference AGB values with corresponding standard deviation, minimum, and maximum.

#### Field data

Field data were collected during November 2012, about six mounts after completion of the field work on the large probability sample. On each of the 30 plots, we registered all trees within a radius limited by the maximum distance measuring range of a Vertex hypsometer [36], which was used to measure the horizontal distance from the plot centre to each tree. The maximum measuring range of the hypsometer varied among the plots due to differences in terrain ruggedness and forest density. The radius distribution among the 30 plots was as follows; 31 m (22 plots), 28 m (2 plots), 26 m (1 plot) and 25 m (5 plots). For each tree with diameter at breast height (*dbh*) larger than 5 cm, scientific name, local name, distance to plot centre and *dbh* was registered. A diameter tape, rather than a calliper, was used to gauge diameters since tree trunks in this forest type tend to be both oval and large in size. The distance was measured from plot center to the front of each tree, and half of the tree diameter was added to get the total horizontal distance. The distance measures enabled us to generate any plot size within the limit of the maximum radius. For this study, we decided to select radii between 7.98 m (200 m^2^) and 30.90 m (3000 m^2^) (Table 4) for further analysis. Three trees (largest, medium and smallest in terms of diameter) per plot were measured for height (*h*) using a Vertex hypsometer.

Precise field coordinates were determined in the centre of each plot by means of differential Global Navigation Satellite Systems (dGNSS). Topcon Legacy 40 channels dual frequency receivers, observing both pseudo-range and carrier phase of the Global Positioning System (GPS) and the Global Navigation Satellite System (GLONASS) were used as rover and base station. The post-processing reports from Pinnacle version 1.0 software [37] indicated an average error of 19 cm for the planimetric coordinates. The error was computed as two times the standard deviations of the corrected single observations reported from Pinnacle output [38].

### Field estimates of AGB

For each plot AGB was estimated by using the local allometric AGB model developed by [39] with both *dbh* and *h* as predictor variables (Eq. 2). Using models with both *dbh* and *h* is reported to moderate the effect of large *dbh*-values on AGB estimates as compared to models with *dbh* only [40-42]. Before calculating AGB, a height model (Eq. 1), was developed using the observations of tree height and corresponding diameters from each plot. A number of model forms for diameter–height relationship [43-48] were tested using non-linear mixed effect approach. Best model fit, judged by the Akaike information criterion (AIC), was obtained using the model form by [46]

1 $$ h=1.3+45.5103\left[ exp\left(-2.7163* exp\left(-0.0354*dbh\right)\right)\right] $$

This model was used to predict height for trees without height measurements. AGB was calculated for individual trees within each plot according to [39] i.e.,

2 $$ AGB=0.4020*{(dbh)}^{1.4365}{(h)}^{0.8613} $$

and then summed to obtain total AGB for the respective plot. The AGB values were finally scaled to per ha values for the different plot sizes (Table 4). The calculated AGB values are henceforth denoted field reference AGB.

### Laser scanner data

ALS data were collected during the period from 19 January to 18 February 2012 using a Leica ALS70 sensor (Leica Geosystems AG, Switzerland) carried by a Cessna 404 fixed-wing aircraft. Mean flying altitude was 800 m above ground covering the entire area of ANR (i.e. wall to wall) at a ground speed of 75 m s^−1^. The scanning rate was 58.6 Hz and the instrument operated at a pulse repetition frequency of 339 kHz with a resulting average pulse density of 10.6 points m^−2^.

Processing of the ALS data started with classification of each ALS echo as ground or vegetation using the progressive irregular triangular network densification method [49] implemented in the TerraScan software [50]. A Triangular Irregular Network (TIN) was created using the ALS echoes classified as ground echoes. The heights above the ground surface were calculated for all echoes by subtracting the respective TIN heights from the height values of all echoes recorded. Up to five echoes were registered per pulse and we used the three echo categories classified as “single”, “first of many”, and “last of many”. The “single” and “first of many” echoes were pooled into one dataset denoted as “first” echoes, and correspondingly, the “single” and “last of many” echoes were pooled into a dataset denoted as “last” echoes.

Several variables were extracted from the ALS data for each of the field plot sizes as described by [51]. For each plot size, height distributions of both first and last echoes were first created. A height threshold of 2.0 m was applied in order to remove the effect of low vegetation and echoes from ground features falsely classified as vegetation. Then, heights at nine percentiles (10^th^, 20^th^, …, 90^th^) of both the first- and last echo distributions were computed to represent canopy height and labeled H_10_.F, H_20_.F, …, H_90_.F (first echoes) and H_10_.L, H_20_.L, …, H_90_.L (last echoes), respectively. Measures of canopy density were also derived for first and last echoes of each plot size. The range between the lowest ALS canopy height (>2 m) and the 95^th^ percentile height was divided into 10 vertical fractions of equal height. Canopy densities were then computed as the proportion of ALS echoes above each fraction to total number of first echoes and labeled D_0_.F (>2 m), D_1_.F, …, D_9_.F. Density variables for the last echo distribution were calculated the same way (relative to total number of last echoes) and labeled D_0_.L, D_1_.L, …, D_9_.L. Furthermore, for both first and last echo height distributions on each plot, the maximum height (H_max._.F and H_max_.L ), mean values (H_mean._.F and H_mean_.L), standard deviation (H_sd_.F and H_sd_.L), coefficient of variation (H_cv_.F and H_cv_.L), and skewness (H_skewness_.F and H_skewness_.L) were computed.

### Data analyses

#### Model development

Multiple linear regression analysis with ordinary least square regression (OLS) was used to develop the statistical models relating the field reference AGB and the predictor variables from the ALS data. To ensure that our modelling approaches met the basic assumptions of OLS, the response variable was transformed to logarithmic scale [11,52], while for the predictors both log transformed and non-transformed variables were used. Separate models with log transformed response and combination of log transformed and non-transformed predictor variables were fitted for each of the plot sizes. We decided to fit separate models (unique variable combinations) for each of the plot sizes, because we wanted the model for each plot size to be the “best” and not be constrained by forcing specific variables into the model.

Variable selection was conducted by using reg-subset in the leaps package in R [53]. The selection of the variables was limited to the best combinations of three or fewer variables in order to avoid multicollinearity among candidate predictors. The preferred models were chosen based on the Bayesian information criterion (BIC) [54]. Adjusted R^2^ was also used for assessing the model fit while multicollinearity was assessed by computing the variance inflation factors (VIF). The VIF values were determined for the individual β parameters. VIF values greater than 10 were regarded as an indication of multicollinearity problems [55].

Log-transformation of the response variable introduces a bias when back-transforming to the arithmetic scale. The model for AGB was therefore adjusted for logarithmic bias according to [56] by adding half of the model mean square error to the constant term before transformation to arithmetic scale.

#### Model validation and accuracy assessment

In order to assess the performance of the models for each plot size, leave-one-out cross–validation (LOOCV) was performed. One field plot at a time was excluded from the dataset, and the model was fitted based on n-1 plots to predict the AGB of the left out plot. Here, n denotes the number of field plots, where i = 1,…, n. Relative root mean square error (RMSE %) and the mean prediction error (MPE%) were used as the measures of reliability and calculated according to

3 $$ \mathrm{RMSE}\%=\frac{\sqrt{{\displaystyle {\sum}_{i=1}^n}{\left({y}_i-{\hat{y}}_i\right)}^2/n}}{\overline{y}}\times 100 $$

4 $$ \mathrm{M}\mathrm{P}\mathrm{E}\%=\frac{{\displaystyle {\sum}_{i=1}^n}\left({y}_i-{\hat{y}}_i\right)/n}{\overline{y}}\times 100 $$

Where *y*_*i*_ and ${\hat{y}}_i$ denote field reference AGB and predicted AGB for plot i, respectively, and $\overline{\mathrm{y}}$ denotes mean field reference AGB for all plots. RMSE% is a good measure of how accurately the model predicts the response and is the most important criterion for fit if the main purpose of the model is prediction [57].

#### Analysis of boundary effects

To analyze the boundary effects we studied how the residual errors of the models were related to the field reference AGB of the trees in an outer buffer zone for different field plot sizes. To archive this, we extracted field reference AGB values for 3 m and 6 m buffers outside the field plots for the plot sizes of 200–1500 m^2^ and 200–1100 m^2^, respectively. We selected the trees with *dbh* > 10 cm and computed AGB per hectare for the largest tree in the buffer and the total AGB per hectare for all trees in the buffer. To obtain the model residual error, we first subtracted the ground reference AGB from the predicted AGB. Then we calculated the ratio between the residuals and the total field reference AGB for the respective plot (i.e., relative residual). Similar ratios between (1) sum of AGB per hectare for all trees in the buffer (SAGB_buffer_) and the field reference AGB for the plot and (2) the maximum AGB per hectare for the largest tree in the buffer (MAGB_buffer,_) and the field reference AGB for the plot were also computed. Two empirical models explaining the variation in the relative residual values using either SAGB_buffer_ or MAGB_buffer_ as explanatory variables were developed. Linear mixed effects (LME) regression using *nlme* add-on package [58] in R was used for model fitting. LME models are linear regression models in which parameters are the sum of the fixed and random effects. In this case the fixed effects were either SAGB_buffer_ or MAGB_buffer_ while plot identity was treated as the random effect. We assumed that each plot will have different random error structures and that the distribution of AGB within these plots is not independent of one another. To test the effect of plot sizes on relative residual, we also fitted the linear regression model which relates relative residuals in absolute form and plot sizes. Absolute value was used because we were interested in the magnitude of the residual regardless of its sign.

#### Efficiency of ALS-assisted AGB estimation

ALS-assisted estimation of AGB within the design-based and model-assisted inferential framework can greatly improve the precision compared to pure field-based estimation. The purpose of this analysis was to quantify the gain in estimated precision of using ALS data relative to a pure field-based estimate for increasing plot sizes.

A basic requirement for validity of design-based inference is the availability of a probability sample [59]. As stated above, the current sample of 30 plots was obtained as a subsample of a probability sample, but the sub-sampling was not conducted according to strict probabilistic principles. However, the sub-sample was selected to resemble important properties of the large probability sample as closely as practically feasible. Thus, a comparison of variances using the current data and assuming a probabilistic design will most likely introduce a bias in the estimators of unknown magnitude. Likewise, when a systematic sample is obtained, it is common to adopt design-based estimators assuming e.g. simple random sampling (SRS) although it is well-known that SRS variance estimators usually are positively biased under systematic sampling. The magnitude of the bias is always unknown for a particular sample because bias is a property of an estimator and not a particular sample. The current analysis was conducted under the assumption that the sample at hand would give a meaningful quantification of the effect of plot size on relative variance estimates. Thus, in the current study we adopted design-based variance estimators assuming simple random sampling and complete cover of ALS data.

Assuming SRS, the variance estimator for the field-based AGB estimate ignoring corrections for finite population is [60].

5 $$ {\hat{V}}_{field} = \frac{{\displaystyle {\sum}_{i=1}^n}{\left({y}_i-\overline{y}\right)}^2}{n\left(n-1\right)} $$

For model-assisted estimation, the variance estimator of the so-called generalized regression estimator is [60].

6 $$ {\hat{V}}_{ALS} = \frac{{\displaystyle {\sum}_{i=1}^n}{\left({\widehat{e}}_i-\overline{e}\right)}^2}{n\left(n-1\right)} $$

where ${\hat{e}}_i={y}_i-{\hat{y}}_i$ is the model prediction residual for plot i and $\overline{e}=\frac{{\displaystyle {\sum}_{i=1}^n}{\hat{e}}_i}{n}$ is the mean residual for all plots. Standard error (SE) was computed as the square root of the variance estimates. Finally, the relative efficiency (RE) of ALS-assisted inventory relative to field-based inventory was calculated for different plot sizes as the ratio of the two variance estimates, i.e.,

7 $$ \mathrm{R}\mathrm{E}=\raisebox{1ex}{${\hat{V}}_{field}$}\!\left/ \!\raisebox{-1ex}{${\hat{V}}_{ALS}$}\right. $$

Values of RE greater than 1.0 indicates higher efficiency of ALS-assisted estimates than field-based estimates for a given plot size. To achieve consistency in the analysis across different plot sizes, the dataset was divided into two major groups. The first group subject to analysis comprised all the 30 plots and allowed consistent analysis of plot size ranging from 200–1900 m^2^. The second group allowing analysis from 200 to 3000 m^2^ consisted of 22 of the plots.

## Abbreviations

ALS: Airborne laser scanning
ABA: Area-based approach
AGB: Aboveground biomass
AIC: Akaike information criterion
ANR: Amani nature reserve
BIC: Bayesian information criterion
dGNSS: differential Global Navigation Satellite Systems
GLONASS: Global Navigation Satellite System
GPS: Global Positioning System
LME: Linear mixed effects
LOOCV: leave-one-out cross–validation
MA: Model-assisted
MAGB_buffer,_: Maximum AGB per hectare for the largest tree in the buffer
MPE: Mean prediction error
MRV: Monitoring, Reporting and Verification
OLS: Ordinary least square regression
REDD+: Reducing Emissions from Deforestation and Forest Degradation in tropical countries
RMSE: Root mean square error
SAGB_buffer_: Sum of AGB per hectare for all trees in the buffer
SAR: Synthetic aperture RADAR
SE: Standard error
SRS: Simple random sampling
TIN: Triangular Irregular Network
UNFCCC: United Nations Framework Convention on Climate Change
RE: Relative efficiency
